# Novel marine metalloprotease—new approaches for inhibition of biofilm formation of *Stenotrophomonas maltophilia*

**DOI:** 10.1007/s00253-023-12781-0

**Published:** 2023-09-27

**Authors:** Marie Kristin Peters, Yekaterina Astafyeva, Yuchen Han, Jascha F. H. Macdonald, Daniela Indenbirken, Jacqueline Nakel, Sanamjeet Virdi, Guido Westhoff, Wolfgang R. Streit, Ines Krohn

**Affiliations:** 1 Department of Microbiology and Biotechnology, Institute of Plant Science and Microbiology, University of Hamburg, Ohnhorststr.18, 22609 Hamburg, Germany; 2 Technology Platform Next Generation Sequencing, Leibniz Institute of Virology, Martinistraße 52, 20251 Hamburg, Germany; 3 Tierpark Hagenbeck, Gemeinnützige Gesellschaft mbH, Lokstedter Grenzstraße 2, 22527 Hamburg, Germany

**Keywords:** Marine habitats, Antimicrobials, Proteases, *Stenotrophomonas maltophilia*, Human health management

## Abstract

**Abstract:**

Many marine organisms produce bioactive molecules with unique characteristics to survive in their ecological niches. These enzymes can be applied in biotechnological processes and in the medical sector to replace aggressive chemicals that are harmful to the environment. Especially in the human health sector, there is a need for new approaches to fight against pathogens like *Stenotrophomonas maltophilia* which forms thick biofilms on artificial joints or catheters and causes serious diseases. Our approach was to use enrichment cultures of five marine resources that underwent sequence-based screenings in combination with deep omics analyses in order to identify enzymes with antibiofilm characteristics. Especially the supernatant of the enrichment culture of a stony coral caused a 40% reduction of *S. maltophilia* biofilm formation. In the presence of the supernatant, our transcriptome dataset showed a clear stress response (upregulation of transcripts for metal resistance, antitoxins, transporter, and iron acquisition) to the treatment. Further investigation of the enrichment culture metagenome and proteome indicated a series of potential antimicrobial enzymes. We found an impressive group of metalloproteases in the proteome of the supernatant that is responsible for the detected anti-biofilm effect against *S. maltophilia*.

**Key points:**

• *Omics-based discovery of novel marine-derived antimicrobials for human health management by inhibition of S. maltophilia*

• *Up to 40% reduction of S. maltophilia biofilm formation by the use of marine-derived samples*

• *Metalloprotease candidates prevent biofilm formation of S. maltophilia K279a by up to 20%*

**Supplementary Information:**

The online version contains supplementary material available at 10.1007/s00253-023-12781-0.

## Introduction

Many marine organisms like jellyfish, sponges, and corals live partly in habitats prone to extreme temperatures, pH values, or salinity (Lordan et al. 2011; Giordano 2021). To survive in such hostile environments, they produce a high diversity of bioactive molecules with unique characteristics, e.g., proteins, polysaccharides, and antioxidants (Lordan et al. 2011; Giordano 2021). Even more evolutionary events in prokaryotic species can be detected in oceans, and these species exhibit an even higher genetic variety (Whitman et al. 1998). There are several studies about pharmaceutically relevant metabolites from marine organisms. Two studies, for example, highlighted that the phylum *Actinobacteria* contains many candidates which produce secondary metabolites with antibacterial, antifungal, and antiviral characteristics (Prashith Kekuda et al. 2010; Manivasagan et al. 2013). Especially the synthesis of antibiotics like oxytetracycline by *Streptomycetes* is of great importance (Petković et al. 2006; Quinn et al. 2020). Furthermore, *Streptomycetes* originating from the soft coral *Sarcophyton convolutum* represent a source of antimicrobial enzymes (El-Gendy et al. 2021). Similar discoveries were made by investigation of sponge-associated bacteria which produce substances like phospholipases or cellulases (Selvin 2008; Shanmughapriya et al. 2009; Santos-Gendelman et al. 2014). In addition, investigations of marine-derived fungi revealed a series of compounds, like polyketides and alkaloids, which act cytotoxically on cancer cell lines (Rateb and Ebel 2011; Shabana et al. 2021). The same effect can be observed by lipopeptides like halobacillin and mixirin from marine *Bacillus* strains (Sansinenea and Ortiz 2011; Mondol et al. 2013).

Metagenomics enables a deep view of the genomes of different microorganisms. After taking an environmental sample, the DNA can be isolated either directly from the material or from enrichment cultures in specific media (Alma'abadi et al. 2015; Popovic et al. 2015). The metagenome DNA can be sequenced and analyzed with different bioinformatics tools and databases. Sequence-based screenings reveal bioactive compounds from habitats of interest (Alma'abadi et al. 2015; Popovic et al. 2015; Robinson et al. 2021). This approach increases the possibility of finding a wider range of extraordinary putative antimicrobial agents in comparison to the cultivation of only one bacterial strain.

In addition, there are studies about enzymes like glycosidases, proteases, and DNases which can degrade extracellular polymeric substances (EPS) and thereby increase the permeability and vulnerability of biofilms for antibiofilm agents (Algburi et al. 2017; Fleming and Rumbaugh 2017; Saggu et al. 2019). Proteases are especially promising because of their ability to disintegrate proteins which are the critical components of the extracellular matrix (Lister and Horswill 2014; Saggu et al. 2019). Aureolysin (Aur), staphopain A (ScpA), and staphopain B (SspB) are examples of proteases that are active on a mature *Staphylococcus aureus* biofilm (Fleming and Rumbaugh 2017; Lister and Horswill 2014). Another point of action represents the extracellular DNA which is important for the first phase of biofilm formation (Whitchurch et al. 2002; Steinberger and Holden 2005; Baslé et al. 2017). For example, *Bacillus licheniformis* produces NucB, an enzyme that inhibits the accumulation of single- and mixed-species biofilms (Rostami et al. 2017).

However, microbial biofilms are increasingly coming into focus because of their relevance to the medical sector. Nearly 80% of all bacterial infections in human beings are attributed to biofilm-producing organisms (Romling and Balsalobre 2012; Fleming and Rumbaugh 2017). Artificial joints, catheters, and other devices which are in direct contact with patients are particularly susceptible to biofilm attachment (Darouiche 2004; Jamal et al. 2018; Zhang et al. 2021). There are a number of bacterial strains that cause such difficulties. The most common ones are *Pseudomonas aeruginosa* and *S. aureus* which are well studied (Driscoll et al. 2007; Lister and Horswill 2014; Guendouze et al. 2017). Another biofilm former has been getting attention: *Stenotrophomonas maltophilia*. This bacterium is aerobic, Gram-negative, rod-shaped, and belongs to the *Gammaproteobacteria* (Hansen 2012; Alio et al. 2020). It is also an opportunistic pathogen and responsible for infections in humans like pneumonia, bacteremia, and endocarditis (Brooke 2012; Abda et al. 2015; Sanchez 2015; Adegoke et al. 2017; Alio et al. 2020). This nosocomial organism forms thick biofilms in the lungs of immunocompromised patients and on medical devices as well.

Due to these serious problems, we chose this candidate for our study. In particular, we worked with the clinical isolate K279a (Crossman et al. 2008; An and Berg 2018). Overall, in this study, we used the approach of enrichment cultures with marine material and sequence-based screenings in combination with metagenomics and proteomics to find potential antimicrobial proteins. We showed that the supernatant of an enrichment culture with a stony coral, *Montipora foliosa*, inhibited biofilm formation most strongly when we incubated it with the pathogen *S. maltophilia* K279a. The fractionation of the supernatant and deeper analysis via LC-MS narrowed it down to a group of active proteases. Thereby our data imply that metalloproteases could be the source of biofilm inhibition. We hypothesize that the fascinating group ultimately destructs the structure of a biofilm and can be applied for therapies against infections with *S. maltophilia* and probably even other strains.

## Material and methods

### Sampling and cultivation

Samples of five coral species were taken at the Hagenbeck Tropical Aquarium (Lokstedter Grenzstraße 2, 22527 Hamburg, Germany). The stony corals *M. foliosa*, *Montipora hodgsoni*, *Seriatopora caliendrum*, *Acropora* sp., and the soft coral *Sinularia brassica* were collected (Supplemental Table S1). For the cultivation, we used a modified Bacto Marine Broth (DIFCO 2216, DSMZ-German Collection of Microorganisms and Cell Cultures GmbH, Braunschweig, Germany, Hansen and Sørheim 1991). The medium was composed of 5.0 g/l Bacto peptone, 1.0 g/l Bacto yeast extract, 0.11 g/l Fe(III) citrate, 19.45 g/l NaCl, 12.40 g/l MgCl_2_ × 6H_2_O, 3.24 g/l Na_2_SO_4_, 1.80 g/l CaCl_2_, 0.55 g/l KCl, 0.16 g/l NaHCO_3_, 0.08 g/lKBr, 0.094 g/l SrCl_2_ × 6H_2_O, 0.022 g/l H_3_BO_3_, 0.004 g/l Na-silicate, 0.0024 g/l NaF, 0.0016 g/l (NH_4_)NO_3_, and 0.010 g/l Na_2_HPO_4_ × 2H_2_O. The pH value was adjusted to 7.6. The corals were broken into smaller pieces, and 0.5 g of each species was used for enrichment cultures in 50 ml Bacto Marine Broth which was inoculated at 28 °C for up to five days. All cultures were centrifuged for 20 min at 4 °C and 5.000 × g. The supernatant was filtered twice through a 0.2-µm syringe filter (CLearLine, Kisker Biotech GmbH & Co. KG, Steinfurt, Germany).

*S. maltophilia* K279a was cultured in LB medium (10 g/l tryptone, 5 g/l yeast extract, and 5 g/l NaCl, pH 7.0) overnight at 37 °C.

### Fluorescence microscopy of *S. maltophilia* biofilms

The structures of the biofilm after treatment with the supernatants of the selected enrichment cultures were examined with the confocal laser scanning microscope (CLSM, Carl Zeiss Microscopy GmbH, Jena, Germany). The samples were prepared in 8-well sterile µ-slides (ibidi GmbH, Gräfelfing, Germany). 100 µl overnight culture of a biofilm former (diluted with the appropriate medium to OD_600 nm_ = 0.05) and 100 µl of the prepared supernatants were added in each well. After incubation at 37 °C for 24 h, the cells were stained with the LIVE/DEAD BacLight bacterial viability kit (Thermo Fisher Scientific, Waltham, MA). Images of the biofilm were taken at the CLSM (confocal laser scanning microscopy) Axio Observer.Z1/7 LSM 800 with airyscan (Carl Zeiss Microscopy GmbH, Jena, Germany) and a C-Apochromat 63x/1.20W Korr UV VisIR objective. In each case, *S. maltophilia* K279a plus Bacto Marine Broth acted as a control. At least three different spots in one sample were chosen for the images. All images were processed with ZEN system software (version 2.3; Carl Zeiss Microscopy GmbH, Jena, Germany). In addition, the cell numbers and the biofilm thickness on 3D images were quantified with the program BiofilmQ (version 0.2.2, https://drescherlab.org/data/biofilmQ/, Drescher Lab, Marburg, Germany, Hartmann et al. 2021).

### Biofilm prevention assay

In order to detect effects on the biofilm density of a common biofilm producer, the biofilm assays in 96-well microtiter plates with Nunclon delta surface (Thermo Fisher Scientific, Waltham, MA) were performed according to Steinmann et al. (2018) with a few modifications. An overnight culture of *S. maltophilia* K279a (Supplemental Table S1, Crossman et al. 2008) was diluted in LB medium (10 g/l tryptone, 5 g/l yeast extract, and 5 g/l NaCl) to OD_600 nm_ = 0.05. 100 µl of the *S. maltophilia* culture was pipetted into 6 wells, and 100 µl of each supernatant recovered from the enrichment cultures was added. After incubation at 37 °C for 24 h, the planktonic cells were removed and the plate was dried at 65 °C for 60 min. In each well, 50 µl of 0.5% crystal violet was added and incubated for 5 min at room temperature. The microtiter plate was washed three times with water and tapped onto a paper towel. Then, the plate was dried at 37 °C for 30 min. After adding 150 µl of 33% acetic acid to each well, the biofilm was thoroughly suspended and the amount of solubilized biofilm was measured in a plate reader at the wavelength of 595 nm.

### Total DNA extraction from liquid samples

After three days of incubation, 4 ml of each enrichment culture was centrifuged for 15 min at 4 °C and 5.000 × g. The DNA of each cell pellet was isolated with the NucleoSpin Microbial DNA Kit (Machery-Nagel, Düren, Germany). The concentration and quality of the DNA samples were measured with a NanoPhotometer (Implen NanoPhotometer, Thermo Fisher Scientific, Waltham, MA).

### Metagenome sequencing, de novo assembly, and binning

DNA extracted from the enrichment culture with *M. foliosa* was used to perform metagenome analysis by next-generation sequencing at the Leibniz Institute of Virology (LIV Hamburg, Germany). Prior to library generation, the concentration of the extracted DNA samples was measured with the DNA High Sensitivity Assay Kit on a Qubit 2.0 Fluorometer (Thermo Fisher Scientific, Waltham, MA). DNA libraries were then generated using the NEBNext® Ultra™ DNA Library Prep Kit for Illumina® (New England Biolabs, Frankfurt am Main, Germany) according to the manufacturer’s instructions. Concentrations of the generated libraries were measured with the DNA HS Assay Kit on the Qubit 2.0 Fluorometer (Thermo Fisher Scientific, Waltham, MA). Additionally, the mean fragment length of each library was determined with the DNA High Sensitivity Chip (Agilent Technologies, Inc., Santa Clara, USA) on an Agilent 2100 Bioanalyzer (Agilent Technologies, Inc., Santa Clara, USA). All samples were diluted to 2 nM, and an equimolar pool was generated. Paired-end sequencing (2 × 150 bp) of the pool was performed on a NextSeq500 (Illumina, San Diego, USA) aiming for ~ 80 million reads per sample. Reads were filtered by the use of a similar host genome: *Montipora efflorescens* (https://www.ncbi.nlm.nih.gov/bioproject/509803) using Bowtie 2 (Langmead and Salzberg 2012) version 2.3.5.1. Unmapped reads were extracted using SAMtools (Li et al. 2009) and finally assembled into scaffolds using SPAdes genome assembler (Prjibelski et al. 2020) version 3.10.1. Only contigs obtained after de novo assembly of length greater than 1 kb were selected for the analysis.

Further analysis of the sequences was done with the help of the Integrated Microbial Genomes (IMG) system (Markowitz et al. 2012, https://gold.jgi.doe.gov/).

### RNA extraction and sequencing

*S. maltophilia* K279a was grown in 24-well microtiter plates with Nunclon delta surface (Thermo Fisher Scientific, Waltham, MA) and treated with supernatants of the enrichment cultures. After incubation at 37 °C for 24 h, the RNA was isolated from the mature biofilm as described below. The biofilm cells were washed out with TRIzol™ Reagent (Zymo Research, Irvine, United States) and centrifuged at a speed of 4,500 × g for 20 min at 4 °C. To extract the RNA, the pellet was mixed with chloroform and incubated for 7 min at room temperature. It was followed by a centrifugation step at 12,000 × g and 4 °C for 12 min. The upper aqueous phase was agitated with 1-ml isopropyl alcohol, incubated for 7 min at room temperature, and centrifuged at 12,000 × g and 4 °C for 8 min. The nucleotides were precipitated by adding 2 ml of 70% (v/v) EtOH to the supernatant and followed by centrifugation at 7500 × g and 4 °C for 5 min. The EtOH was removed, and the pellet was dried in a vacuum concentrator (SpeedVac, Thermo Fisher Scientific, Waltham, MA) and resuspended in 90-µl diethyl pyrocarbonate (DEPC)-treated water. The DNA was removed with a DNase kit (RNase-free DNase set, Qiagen, Hilden, Germany). Afterward, the sample was mixed with 200 µl RNase-free water and 200 µl PCI (50% phenol/48% chloroform/2% isoamyl alcohol) and loaded on a Phase Lock reaction tube (5PRIME Phase Lock Gel, Quantabio, Beverly, MA). After centrifugation at 11,000 × g for 10 min at 4 °C, the supernatant was transferred to a new reaction tube. The RNA was precipitated by adding 25-µl 3 M NaAc (pH 5.2) and 1-ml ice-cold 96% (v/v) EtOH. The precipitation of the RNA took place overnight. The sample was centrifuged at 12,000 × g for 30 min at 4 °C, the supernatant was discarded, and the pellet was dried in the vacuum concentrator. Finally, the RNA was dissolved in 50-µl DEPC-treated water and was stored at − 70 °C. The concentration and quality of the RNA were measured by a NanoPhotometer (Implen NanoPhotometer, Thermo Fisher Scientific, Waltham, MA) and verified on a 0.8% agarose gel. Optionally, another DNase treatment or a clean-up with the RNA Clean and Concentrator™ Kit (Zymo Research Europe, Freiburg, Germany) was performed. The samples were processed by Eurofins Genomics Europe Sequencing GmbH (Constance, Germany), where the RNA was assessed for quality check (QC). The RNA integrity number (RIN) for all samples was ≥ 8. Strand-specific cDNA library preparation from polyA enriched RNA (150 bp mean read length) and RNA sequencing was performed using the genome sequencer Illumina HiSeq technology in NovaSeq 6000 S4 PE150 XP sequencing mode. For further analysis, fastq-files were provided.

### Processing and analysis of RNA-seq reads

RNA-seq analysis was performed using PATRIC, the Pathosystems Resource Integration Center (http://www.patricbrc.org). Trim Galore 0.6.5dev was used to remove adapters (Phred quality score below 20) (Krueger 2012). RNA-Seq data was processed by the tuxedo strategy (Trapnell et al. 2012). All genes were selected with |log2 (fold change) |≥ 1.5. The differentially expressed gene (DEGs) dataset was collected and used for further analysis. The volcano plot of the distribution of all DEGs was generated using A Shiny app ggVolcanoR (Mullan et al. 2021).

### Fractionation of the supernatant via fast protein liquid chromatography (FPLC)

FPLC (Amersham Biosciences ÄKTA FPLC system, Marshall Scientific, Hampton NH) was used for further analysis of the supernatant. The enrichment culture of *M. foliosa* was centrifuged at 5,000 × g for 20 min at 4 °C, and the supernatant was filtered through a 0.2-µm syringe filter (CLearLine, Kisker Biotech GmbH & Co. KG, Steinfurt, Germany). Afterwards, the supernatant was concentrated in the vacuum centrifuge (SpeedVac, Thermo Fisher Scientific, Waltham, MA) and filtered through a syringe filter again. The prepared sample was loaded on a Superdex® 200 10/300 GL gel filtration column (Sigma-Aldrich Chemie GmbH, Taufkirchen, Germany) with potassium phosphate buffer pH 7.0 as a moving fluid. The fractionation occurred in 1-ml steps with a flow rate of 0.75 ml/min, and the UV spectrum was analyzed with the UNICORN™ system control software (https://www.cytivalifesciences.com).

### Proteome analysis

The supernatant of the *M. foliosa* culture and a control with Bacto Marine Broth were sent to NH DyeAGNOSTICS GmbH (Halle, Germany) for deep analysis of the proteome according to Shevchenko et al. (2007). In the first step, reduction and alkylation of the samples and a tryptic digest were performed. The measurement of tryptic peptides by electrospray ionization mass spectrometry coupled with liquid chromatography (nano-RP-HPLC-ESI-QTOF-MS/MS; Waters nano ACQUITY UPLC coupled with G2-Si; Waters, Milford, MA) followed. The acquired MS data were processed with Mascot Distiller software (Matrix Science, http://www.matrixscience.com/). Provided sequence data, Swissprot/UniProt (universal protein database, https://www.uniprot.org, The UniProt Consortium 2021), and database of *Bacillus cereus* were used with different filters (trypsin, 2 missed cleavage sites, carbamidomethyl modification (Cys), and oxidation (Met)). Unassigned spectra were analyzed using Peaks software (BSI, Baden, Switzerland). The results were provided as filtered (filter: min. 3 detected peptides per protein), unfiltered, and de novo peptides (generated using Peaks; BSI). Raw datasets for the proteome analyses are available via PanoramaWeb (https://panoramaweb.org) under the JobID 158530 and JobID 158529.

### Statistical analysis

For the determination of the significance of certain data, a statistical test was used. The number of living and dead cells, the biofilm thickness, and the biofilm density were subjected to a paired sample *t*-test, and the *p* value was assigned to determine if the two samples were significantly different from each other.

## Results

### High impact of bacterial marine enrichment culture on the biofilm formation of *S. maltophilia* K279a

**Fig. 1:**
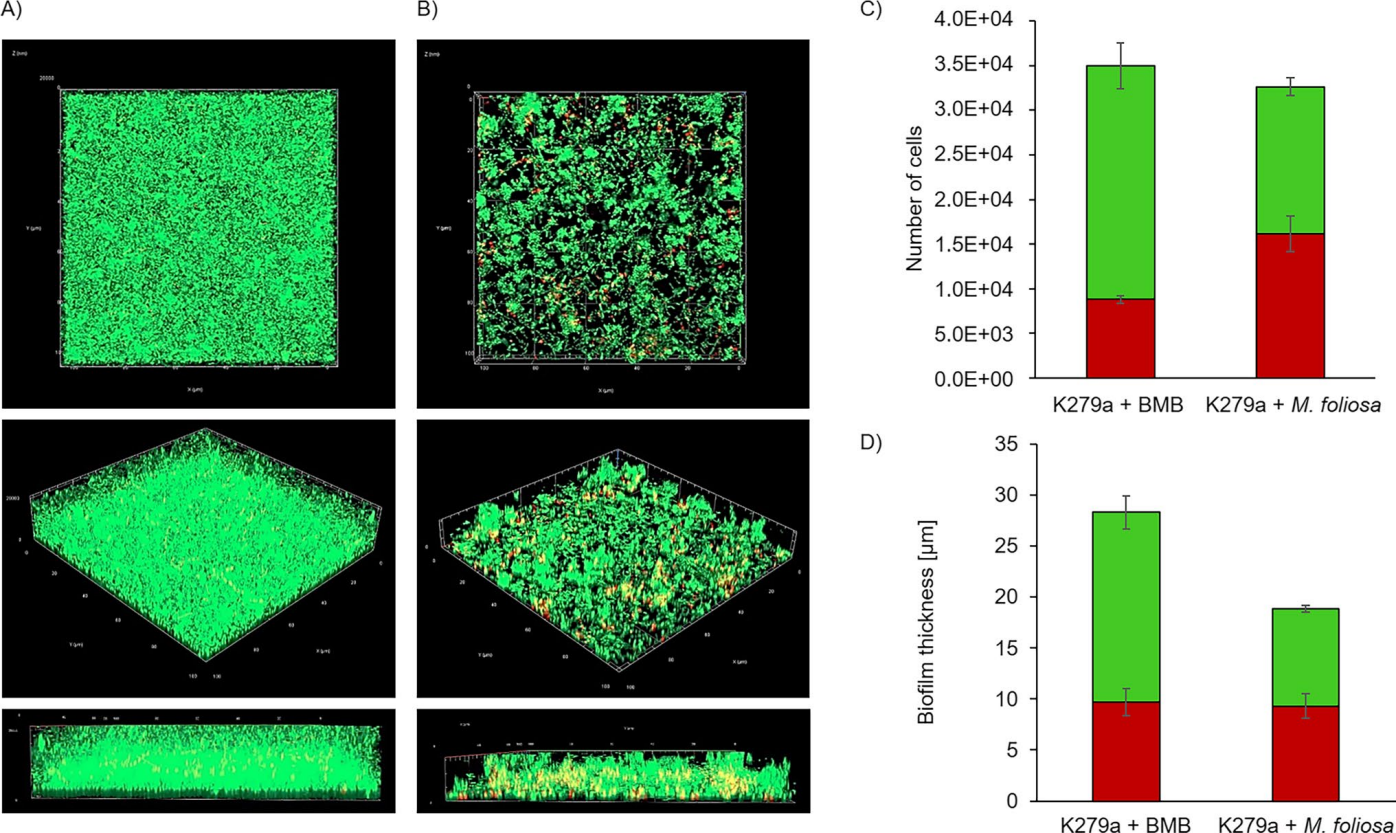
Confocal microscopic analysis of *S. maltophilia* K279a biofilms. Cells were grown under static conditions for 24 h in LB medium and treated with different supplements (BMB, *M. foliosa*). Stained with LIVE/DEAD staining, green: living cells, red: dead cells, and investigated with CLSM. Images represent an area of 100 µm by 100 µm of the biofilm. **A** Control: K279a + Bacto Marine Broth (BMB), and **B** K279a + sterile filtered supernatant of an enrichment culture of *M. foliosa*. **C** The total number of cells and **(D)** biofilm mean thickness are represented in green (living cells) and red (dead cells). Calculation of cell numbers and density of the biofilm were performed with MATLAB/BiofilmQ (https://drescherlab.org/data/biofilmQ/docs/). Statistical analyses were subjected to a paired sample *t*-test, and the *p* value was referred to define if the two samples are significantly different from each other. Significant biofilm reduction marked by stars (significance level *p* value ≤ 0.05). The number of dead cells was significantly higher with a *p* value of 0.01 in the sample with *M. foliosa* compared to the control, and the biofilm thickness was significantly reduced with a *p* value of 0.04. The data are mean values of at least three replicates. The error bars indicate sample standard deviations

To identify enzymes interfering with microbial biofilm formation, we tested selected enrichment cultures of marine corals within a preliminary biofilm prevention assay (Supplemental Table S1 and Supplemental Fig. S1). The model organism *S. maltophilia* K279a was treated with the filtered supernatant from the cultures. The biofilm density was measured after 24 h of incubation. The best results were observed with a supernatant obtained from a three days *M. foliosa* enrichment culture. This culture supernatant significantly reduced the biofilm density by about 40% (± 16%) in comparison to the control (Supplemental Fig. S1). For a deeper investigation, we analyzed the biofilm structure of K279a via CLSM and determined the cell numbers and biofilm thickness with BiofilmQ (Hartmann et al. 2021, Fig. 1). The appearance of the biofilm treated with the supernatant differed clearly from the control with Bacto Marine Broth. It was less dense and partially porous. The total number of cells in the control was 34,961, in detail 26,139 living and 8,822 dead cells. In comparison, the *M. foliosa* sample showed 16,457 living and 16,181 dead cells (Fig. 1C). The number of dead cells in this sample was significantly higher than in the control with a *p* value of 0.01. The analysis of the biofilm thickness also indicated the reduction effect of the supernatant on K279a. The biofilm treated with the *M. foliosa* sample was significantly reduced by about 33% with a *p* value of 0.04 (Fig. 1D). These results indicate that the supernatant from the enrichment culture of *M. foliosa* consortium might contain certain biofilm-reducing and/or antimicrobial molecules.

### Metagenome-based microbial community analyses of *M. foliosa*

**Fig. 2:**
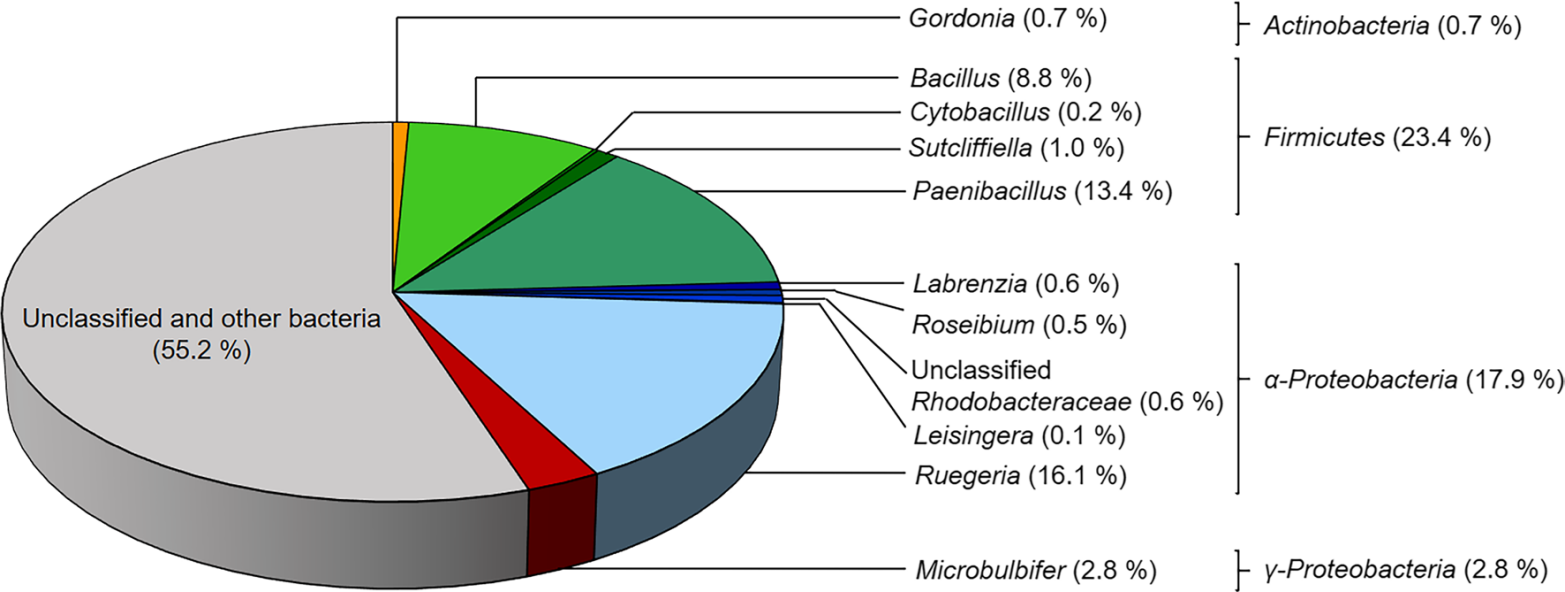
Phylogenetic analysis of microbial communities from an enrichment culture with *M. foliosa*. Representation of the percentage distribution of different genera within the metagenome. The affiliation based on the Integrated Microbial Genomes and Microbiomes (IMG, 3300038501) database

To get an indication of which biomolecules are responsible for the detected effects, the metagenome of the microbial community from *M. foliosa* was sequenced via Illumina*®* NextSeq500. Raw data contained 83,679,656 sequences with lengths of about 151 bp and a GC content of 42%. The assembled metagenome was analyzed with the Integrated Microbial Genomes and Microbiomes database (IMG, https://gold.jgi.doe.gov/, Markowitz et al. 2012) and covered 42,166,744 bp with a GC count of 48.39% and 47,903 protein-coding genes (Supplemental Table S2). To ascertain which organisms are the key players in this consortium, we investigated the phylogenetic distribution (Fig. 2). In total, the number of different species within the metagenome was limited to 55. The most abundant bacteria phylum was *Firmicutes* with 23.4%, followed by *Alphaproteobacteria* (17.9%), *Gammaproteobacteria* (2.8%), and *Actinobacteria* (0.7%). *Firmicutes* were split up into four genera, *Paenibacillus* (13.4%), *Bacillus* (8%), *Sutcliffiella* (1.0%), and *Cytobacillus* (0.2%). The *Alphaproteobacteria* were represented by *Ruegeria* (16.1%), *Labrenzia* (0.6%), *Roseibium* (0.5%), *Leisingera* (0.1%), and 0.6% unclassified *Rhodobacteraceae.* In addition, 2.8% *Microbulbifer* (*Gammaproteobacteria*) and 0.7% *Gordonia* (*Actinobacteria*) were found.

### Antimicrobial potential of marine communities from *M. foliosa*

For further investigations, we used the COG (Clusters of Orthologous Groups) databases (https://www.ncbi.nlm.nih.gov/research/cog-project/) and performed a functional analysis of the metagenome. About 25% of all key features were represented by amino acids, carbohydrate, and energy metabolism. In addition, membrane transport and metabolism of cofactors and vitamins were highlighted with nearly 13% (Table 1). Signal transduction was represented with 4%, nucleotide metabolism with nearly 4%, and lipid metabolism and translation mechanisms with 3%. The metabolism of other amino acids, replication, repair, and xenobiotics biodegradation was observed at about 2%. The following key features were all represented with 1%: biosynthesis of secondary metabolites, cell motility, drug resistance, folding, sorting, degradation, glycan biosynthesis, and metabolism of terpenoids and polyketides.

**Table 1:** Key features observed in the microbial communities from *Montipora foliosa* using IMG data analysis

Trait	% of all hits
Amino acid metabolism	10.02
Biosynthesis of other secondary metabolites	1.09
Carbohydrate metabolism	8.94
Cell motility	1.57
Drug resistance	1.04
Energy metabolism	5.96
Folding, sorting, and degradation	1.79
Metabolic pathways of global and overall metabolism	27.01
Glycan biosynthesis and metabolism	1.29
Lipid metabolism	3.08
Membrane transport	7.33
Metabolism of cofactors and vitamins	5.64
Metabolism of other amino acids	2.62
Metabolism of terpenoids and polyketides	1.59
Nucleotide metabolism	3.87
Replication and repair	2.04
Signal transduction	4.16
Translation	3.1
Xenobiotics biodegradation and metabolism	2.22
Others	5.64
Total	100

In the next step, we focused on the overall enzymes potentially involved in anti-biofilm activity (Table 2). Potential anti-biofilm enzymes were involved in hydrolysis of extracellular polymeric substances (EPS), quorum quenching mechanisms, and cell wall degradation processes. The COG-based search showed a range of diverse biomolecules, described for potential anti-biofilm mechanisms in literature. In total, we detected 49 chelatases, 175 deacetylases, 224 deaminases, 195 decarboxylases, 181 endonucleases, 472 lyases, 10 lysozymes, and 14 polyketide synthases. However, one group accounted for most of these enzymes, namely, 515 proteases. Beyond that, we found many potential quorum quenching enzymes, such as 148 acylases, 39 lactonases, and 605 oxidoreductases.

**Table 2:** Key features of putative antimicrobial and quorum quenching active enzymes observed in the bacterial communities from *Montipora foliosa* using a COG-based analysis. Data shown in total number of hits per 100 Mb

Enzymes	Total number of hits
Chelatases	49
Magnesium chelatases	15
Deacetylases	175
Deaminases	224
Cytosine/adenosine deaminase-related metal-dependent hydrolases	33
Decarboxylases	195
Arginine decarboxylases	14
Endonucleases	181
Restriction endonucleases	22
Lyases	472
Lysozymes	10
Polyketide synthases	14
Proteases	515
Serine proteases	111
Metalloproteases	18
Quorum quenching	
Acylases	148
Lactonases	39
Dienelactone hydrolases	16
6-Phosphogluconolactonases	20
Oxidoreductases	605
Fe-S oxidoreductases	51
FAD-dependent oxidoreductases	32

### Transcriptome analysis indicated highly active genes of biofilm formation and stress response

**Fig. 3:**
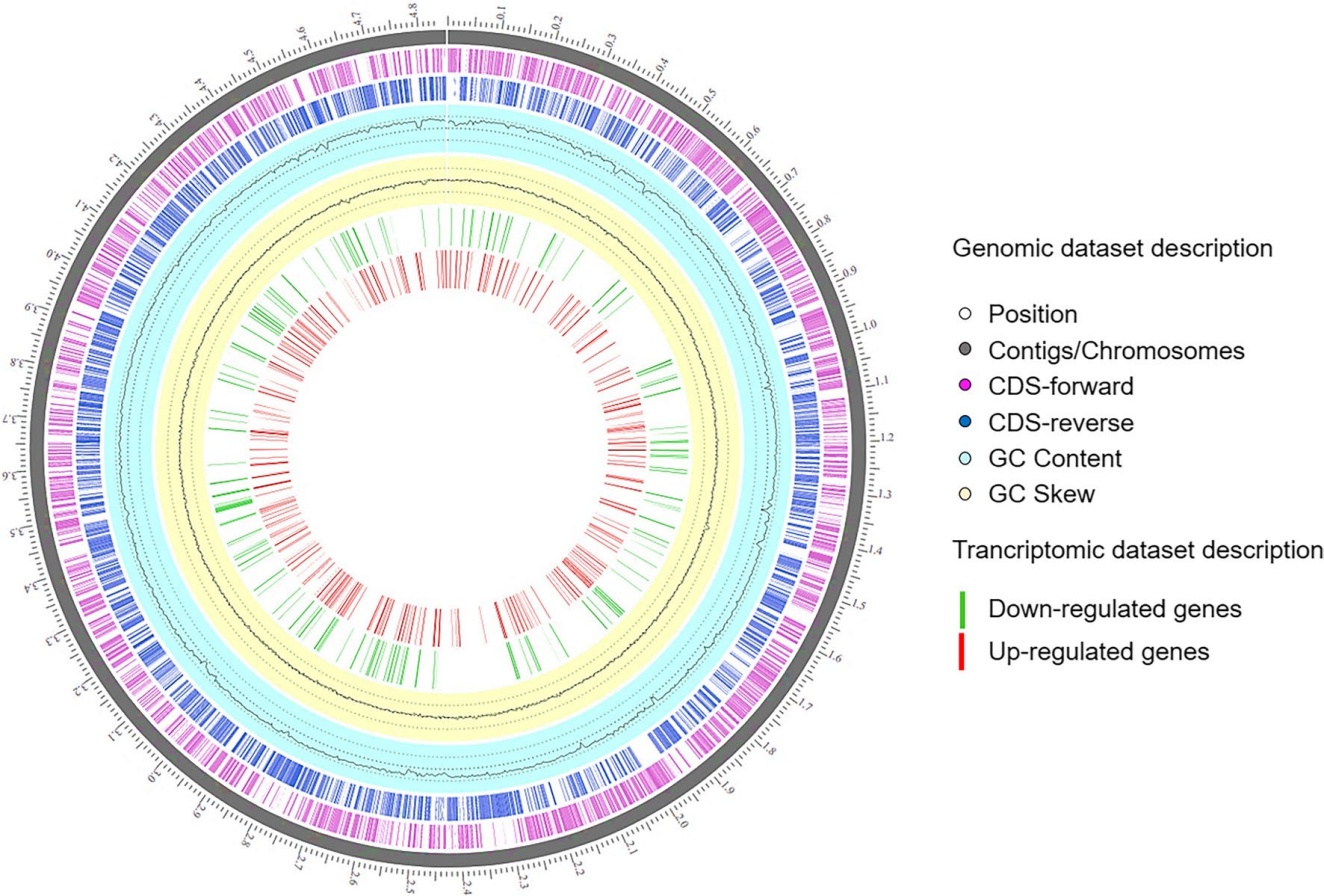
Transcriptome analysis and circular genome mapping of *S. maltophilia* K279a after treatment with supernatant of *M. foliosa* enrichment culture (GenBank: AM743169.1). Moving inward, the subsequent two rings show coding sequences (CDSs) in forward (magenta) and reverse (blue) strands. Cyan and yellow plots indicate GC content and a GC skew [(GC)/(G + C)]. Transcriptomic dataset description; red: upregulated genes, green: downregulated genes

**Fig. 4:**
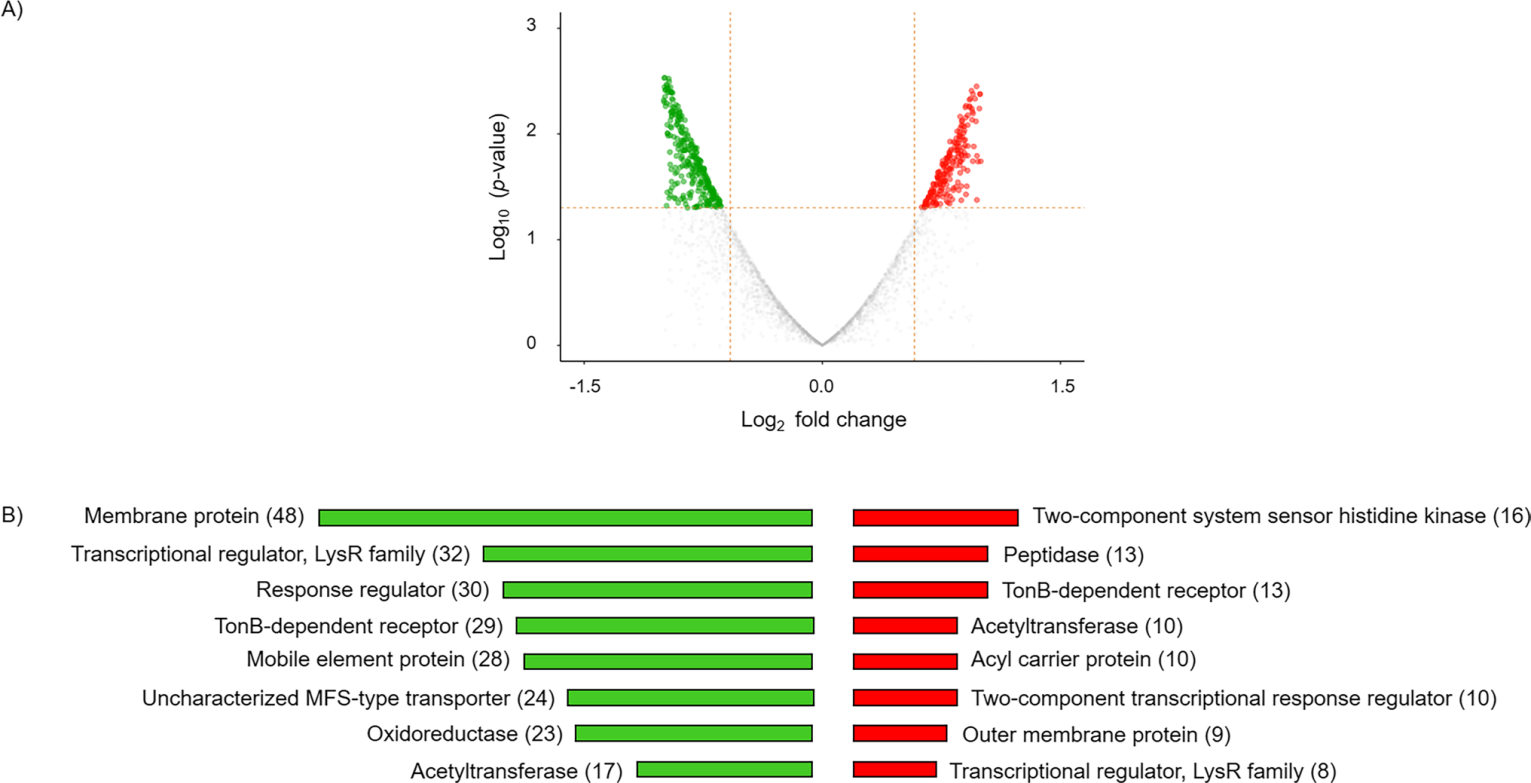
Differentially expressed genes (DEGs) in *S. maltophilia* K279a in response to *M. foliosa* enrichment culture compared with control dataset, all genes were selected with |log2 (fold change) |≥ 1.5. **A** Volcano plot is highlighting the DEGs in *S. maltophilia* K279a, *x*-axis: log_2_, large-scale fold changes; *y*-axis: –log10 of the *p* value showing the statistical significance. Each point corresponds to one gene. The points above the vertical and horizontal dotted lines represent log_2_FC ≥ 0.58 and *p* value < 0.05. A volcano plot was generated using A Shiny app ggVolcanoR.** B** Functional description of highly active up- (red) and down- (green) regulated genes of *S. maltophilia* K279a in response to *M. foliosa* enrichment culture

To unravel the potential inhibitory mechanism behind the strong biofilm, we investigated the transcriptome of *S. maltophilia* K279a. The aim of this analysis was the identification of genes involved in this process and to clarify the effect of *M. foliosa* supernatant on gene level as well. The next-generation sequencing (NGS) of the biofilm mRNA resulted in 8,895,226 sequences with an average length of 149 bp and a GC content of 62.50% (Supplemental Table S3). The circular genome mapping of *S. maltophilia* K279a was generated using the Circular Genome Viewer tool within PATRIC, the Pathosystems Resource Integration Center (http://www.patricbrc.org) (Fig. 3). The up- and downregulated genes were presented in the inner rings in red and green, respectively (Fig. 3), and summarized in Supplemental Table S4. Their significance has been plotted in the volcano plot (Fig. 4A).

Data depicted in Fig. 4 and Supplemental Table S4 demonstrate clearly that K279a reacted to the supernatant of the *M. foliosa* enrichment culture. There was evidence of biofilm building but also of stress response in general. The distribution of gene expression between *S. maltophilia* K279a incubated with the supernatant of *M. foliosa* enrichment culture and control sample is represented by the volcano plot (Fig. 4A). The volcano plot was constructed to compare the two groups using ggVolcanoR. A total of 1,530 differentially expressed genes (DEGs) were identified from the dataset (Fig. 4A). Among them, 612 and 918 genes were upregulated and downregulated, respectively, between the two groups according to their log_2_FC and *p* values. The strongest and most significantly differentially regulated genes were 32 counts of downregulated and 8 counts of upregulated genes for transcriptional regulators in the LysR family (Fig. 4B). Besides that, there were 13 gene counts for peptidases, 10 for acyl carrier proteins, and 10 for two-component transcriptional response regulators, which were upregulated. Interestingly, several counts for TonB-dependent receptors (13 upregulated and 29 downregulated) could be detected. The largest group of upregulated genes was represented by two-component system sensor histidine kinases (16). The downregulated genes were dominated by 48 counts of membrane proteins. In contrast, there were only a few outer membrane proteins (9), which were upregulated. In addition, the analysis of downregulated genes resulted in response regulators (30), mobile element proteins (28), uncharacterized MFS-type transporter (24), and oxidoreductases (23). The group of acetyltransferases was represented by 17 counts of downregulated and 10 counts of upregulated genes. More details about all up- and downregulated genes related to the response of K279a to the *M. foliosa* culture have been listed in Supplemental Table S4. We could identify genes linked to defense mechanisms, carbohydrate, coenzyme, ion and lipid transport and metabolism, methionine biosynthesis, oxidative stress-related mechanisms, repair mechanisms, signal transduction, transporter, efflux pumps, and secretion systems. In detail, two genes coding for the metal resistance proteins Acr3 and CzcD and one gene coding for an antitoxin to RelE-like translational repressor toxin were highly upregulated. In addition, two genes for major facilitator superfamily (MFS) transporter systems and one gene for an autotransporter related to pathogenesis were upregulated as well. The deeper analysis of antimicrobial and defense mechanisms revealed downregulated antimicrobial enzymes like FAD-binding monooxygenase, endonucleases, and exonucleases. In addition, genes within inorganic ion transport and metabolism mostly covered iron acquisition, arsenic, and heavy metal resistance, which are known to be connected with biofilm formation (Farasin et al. 2017; Hoeft et al. 2010; Kang and Kirienko 2018; Teitzel and Parsek 2003). A series of genes connected to signal transduction and regulation mechanisms were downregulated. These genes included transcriptional regulators, chemotaxis genes, and iron-sulfur cluster proteins that act as a sensor of the environment and enable the organism to adapt to the prevailing conditions (Crack et al. 2012; Tout et al. 2015).

### Secretome-based analysis provided a large group of hydrolases

We further investigated the supernatant using proteomic tools to verify the metagenome and transcriptome-based results. The investigation of the total supernatant proteome resulted in 338 peptides with masses between 31 and 159 kDa (Supplemental Table S5). In general, we found hydrolases, including proteases, alpha-amylases, glutathione hydrolase proenzymes, chitosanases, oligopeptide-binding proteins, pectate lyases, glucuronoxylanases, and phosphoesterases. However, the most abundant enzymes were proteases, in particular, 77 metalloproteases, 46 serine proteases, 32 neutral proteases, 17 cell wall-associated proteases, and 13 extracellular proteases. Nearly all hydrolases detected in the proteome originated from *Bacillus* strains. This detection corresponded with our phylogenetic analysis of the metagenome (Fig. 2). About 9% of all classified bacterial strains in the enrichment culture belonged to the genus *Bacillus*.

The fractionation with the FPLC system refined the search for the responsible enzymes in the supernatant of the *M. foliosa* enrichment culture. The concentrated sample was collected into 33 units, and the contained proteins were separated according to their size. During this process, the large molecules were eluted from the column first and the small ones last. Afterwards, all fractions were analyzed and 8 fractions that contained putative proteins (Supplemental Fig. S2 A) were detected. To verify the activity, biofilm prevention assays with *S. maltophilia* K279a were performed and shown in Supplemental Fig. S2 together with the undiluted supernatant. All samples were compared to the strain with potassium phosphate buffer (pH 7.0) which acted as a negative control. Astonishingly, the whole supernatant could reduce the biofilm density about 40%, just as the commercially acquired protease (subtilisin A) from *B. licheniformis* (P5380, Sigma-Aldrich, Merck KGaA, Darmstadt, Germany). The fractions 23 and 24 also significantly decreased the biofilm by about 20%, in contrast to the negative control (Supplemental Fig. S2 B).

The proteome analysis of these highly interesting active fractions led to significant sequences of a putative microbial protease with a mass of 100 kDa and a sequence coverage of 0.74 (Supplemental Table S6). A search with the data resource UniProt (universal protein database, https://www.uniprot.org, The UniProt Consortium 2021) and NCBI (National Center for Biotechnology Information, https://www.ncbi.nlm.nih.gov/, Sayers et al. 2021) resulted in the protease ColA, with a sequence identity of 99.89%. This enzyme is a highly active peptidolytic and collagenolytic protease originating from *B. cereus* (Abfalter et al. 2016).

### Novel potential metalloproteases-phylogenetic assignment and proposed mechanism

**Fig. 5:**
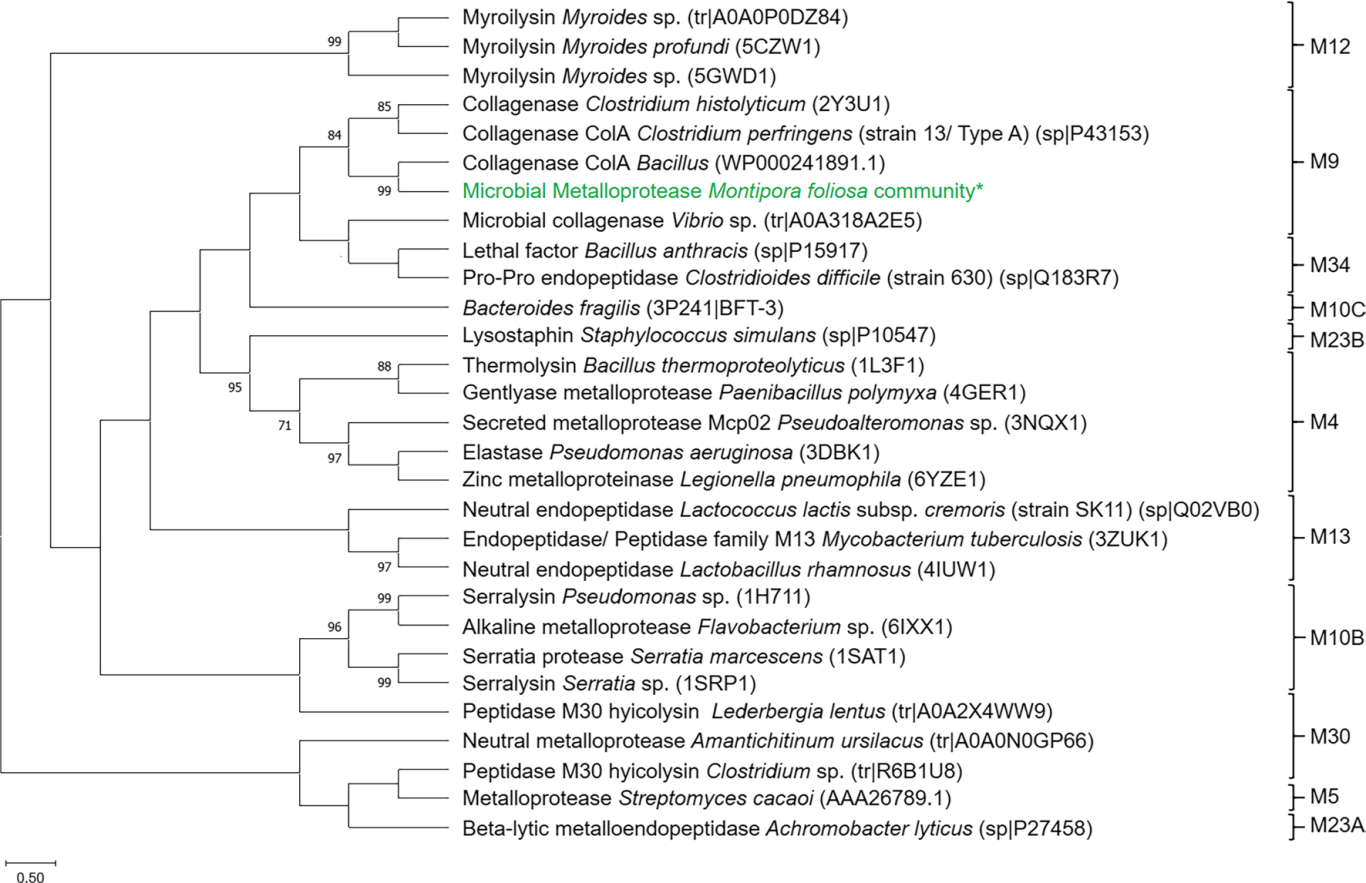
Phylogenetic tree of bacterial extracellular metalloproteases (BEMPS) grouped in MEROPS families. The phylogenetic tree was constructed with MegaX (Kumar et al. 2018) using the maximum likelihood method and JTT matrix-based model (Jones et al. 1992). The bootstrap consensus tree deviates from 1000 replicates (Felsenstein 1985) after multiple alignments with T-Coffee (https://tcoffee.crg.eu/, Notredame et al. 2000). The percentage of bootstrap resamplings ≥ 70 is illustrated on the branches. The scale bar represents the expected number of changes per amino acid position. This classification of metalloproteases is based on Wu and Chen (2011). *The predicted microbial metalloprotease from the bacterial community of *M. foliosa* is integrated into the MEROPS family M9

The proteome investigation of the two fractions (fractions 23 and 24), as described in the previous section, revealed a potential metalloprotease (Fig. 5) with 38 α-helices and 17 β-sheets and a catalytic Zn^2+^ in the active site (Abfalter et al. 2016). The phylogenetic analysis indicated that this protein belongs to the MEROPS (MEROPS database, http://merops.sanger.ac.uk) family M9 of bacterial extracellular metalloproteases (BEMPs) (Fig. 5, Wu and Chen 2011). The M9 family contains, besides ColA, metalloproteases from *Vibrio* sp. or *Clostridium histolyticum* (Fig. 5). In total, 11 different groups or subgroups form this class of metalloproteases. The phylogenetic tree (Fig. 5) shows a few examples for each group and assigns the enzyme detected in this study.

## Discussion

In the last years, biofilm-forming microorganisms, like *P. aeruginosa* and *S. maltophilia*, have come more into focus because of their ability to cause serious diseases. *S. maltophilia* strains have evolved high resistance to common antibiotics, e.g., quinolones and cephalosporins (Looney et al. 2009; Sanchez 2015). For this reason, there is a need for new biomolecules and enzymes to attack the biofilm of pathogens and stop their spreading. However, to date, there are only a few studies describing effective antimicrobials against *S. maltophilia*. In this study, we addressed this current topic and provided a strategy to disrupt biofilm formation.

Our preliminary biofilm assays led to a highly interesting enrichment culture supplemented with parts of the stony coral *M. foliosa*. One remarkable and interesting fact is that corals like *M. foliosa* live in symbiosis with microorganisms which are important for the health and resilience of these marine invertebrates (Shnit-Orland and Kushmaro 2009; Peixoto et al. 2017). There are studies about bacteria living in the mucus that produce substances to compete with other microorganisms (Shnit-Orland and Kushmaro 2009; Pereira et al. 2017; Peixoto et al. 2017). The overall investigations of the metagenomic dataset of our *M. foliosa* enrichment culture showed that *Firmicutes* was the dominant phylum. Interestingly, several candidates belonging to the genus *Bacillus* showed antimicrobial activity by secretion of various metabolic components. For example, a *Bacillus firmus* culture extract could reduce the biofilm of methicillin-resistant *S. aureus* (MRSA) about 83% (Ghosh et al. 2022). With bioinformatical analysis of our datasets, we detected numerous potential antimicrobial agents, especially proteases, endonucleases, chelatases, and quorum quenching active enzymes. With the additional tool of transcriptome analysis, we were able to investigate the pathogen and its reaction to the added supplements in detail. These datasets provided evidence that the supernatant of the *M. foliosa* enrichment culture strongly influenced *S. maltophilia* K279a biofilm formation and induced stress response. Outstanding was the set of upregulated genes, like the transcriptional regulator LysR, which act as a virulence factor in pathogenic microorganisms and is crucial for biofilm formation and protease production (Wang et al. 2021; Islam et al. 2021). Besides, there were gene counts for TonB-dependent receptors, which are responsible for the uptake of iron-binding siderophores (Pawelek et al. 2006; Noinaj et al. 2010). Iron acquisition is indispensable for the transition of a pathogen from planktonic growth to biofilm building (Kang and Kirienko 2018; Zhang et al. 2021; Berlutti et al. 2005). A study of the transcriptomes of seven different *S. maltophilia* isolates also validated the upregulation of genes coding for TonB-dependent receptors (Alio et al. 2020). Furthermore, we identified several gene counts for sensor histidine kinases and peptidases. These two enzyme groups are involved in the adaptation to changing conditions and stress response mechanisms, respectively (Khorchid and Ikura 2006; Culp and Wright 2017). We detected the upregulation of a gene coding for the membrane protein Acr3. This permease acts as a metalloid antiporter and is thus responsible for arsenite resistance (Wawrzycka et al. 2017; Aaltonen and Silow 2008). Appropriately, the major facilitator superfamily (MFS) transporter system could be identified, known for the efflux of arsenicals as well (Garbinski et al. 2019). The CzcD protein, which was also highly upregulated, acts in a similar functional way. It exports heavy metals, like cobalt, zinc, and cadmium, from the cytoplasm into the periplasm (Anton et al. 1999; Sullivan et al. 2021). Additionally, there was proof of a toxin-antitoxin system for preventing toxicity and probably building persister cells (Wang and Wood 2011; Kasari et al. 2013).

However, in general, a biofilm protects the bacterial cells against outside influences like chemicals and predators. Additionally, the physical barrier increases the antibiotic resistance up to 1000-fold (Fleming and Rumbaugh 2017; Rogers et al. 2010). To get access to the bacterial strains inside the film, the structures of the extracellular matrix must be attacked. Studies like Elchinger et al. (2014) showed proteases can be applied in an early stage of biofilm formation. Extracts of a flavourzyme and a neutrase were effective against *Staphylococcus epidermidis* and partly against *S. aureus* biofilm. Especially the degradation of matrix proteins, like protein A, fibrinogen-binding proteins, and clumping factor B, and associated destabilization of the biofilm is a target-oriented strategy (Lister and Horswill 2014; Saggu et al. 2019). Matrix metalloproteases (MMPs) are known to disintegrate parts of human extracellular matrixes (Pardo et al. 2008; Rowan et al. 2008). Glycoproteins, like collagen and elastin, are points of action (Cui et al. 2017). For example, the bacterium *Microbacterium* sp. SKS10 secretes a metalloprotease able to remove *S. aureus* biofilm (Saggu et al. 2019). There is evidence for a human matrix metalloprotease, more precisely a collagenase, that prevents and degrades *Enterococcus faecalis* biofilm effectively (Kumar et al. 2019).

**Fig. 6:**
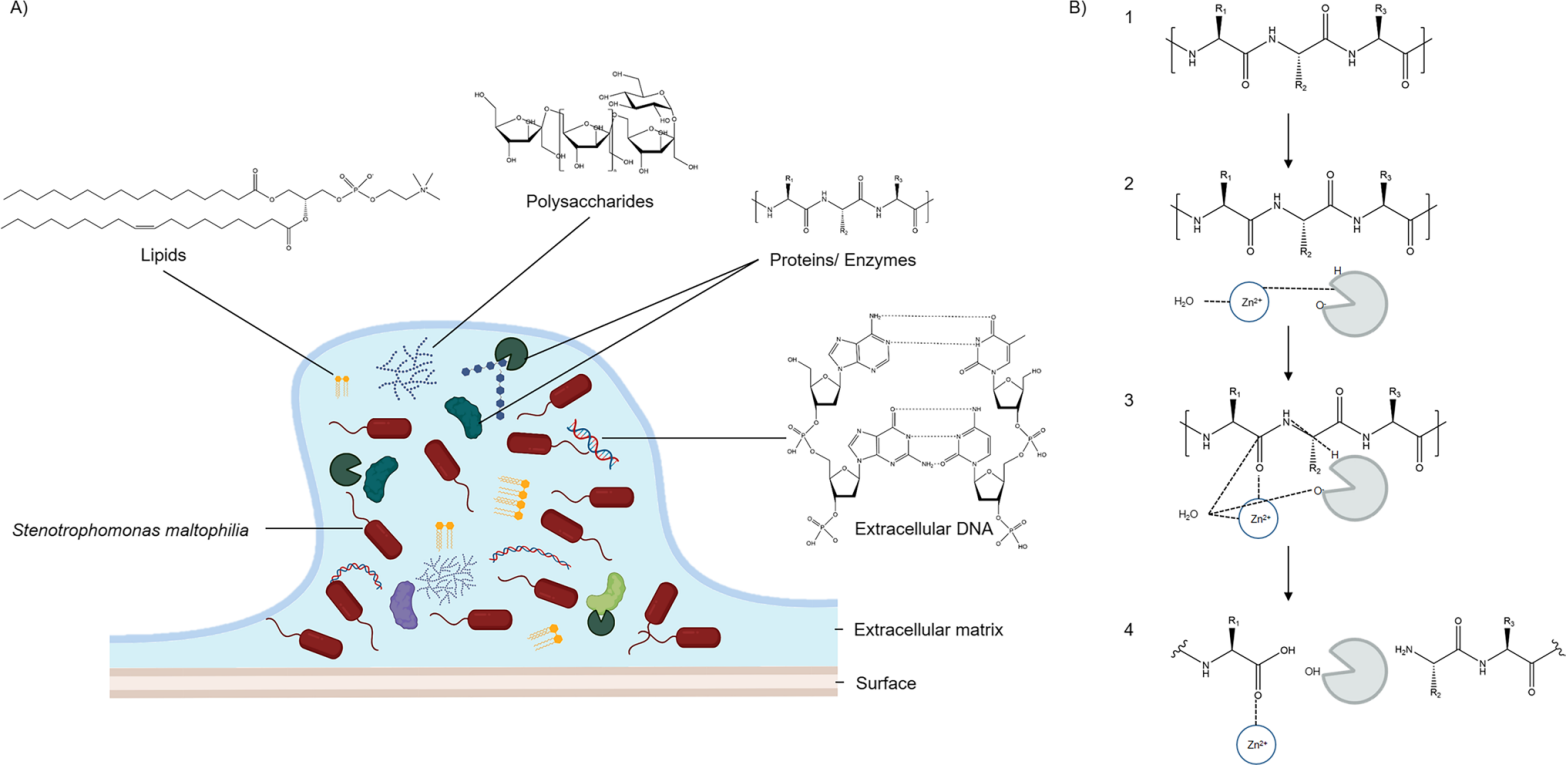
Schematic model of *S. maltophilia* biofilm including compounds and proposed mechanism of metalloproteases. **A** Model of *S. maltophilia* K279a biofilm structure including selected extracellular polymeric substances of lipids (structure: phosphatidylcholin), polysaccharides (structure: levan), proteins/enzymes, and extracellular DNA. **B** General mechanism of metalloproteases (modified, based on Hofmann 1985; Elsässer and Goettig 2021). (1) General peptide structure with R1-R3 representing side chain specifying the amino acid, serving as substrate for the metalloprotases. (2) A water molecule is kept in place by a zinc-(II)-cation which bonded on histidine of the metalloprotease. The endometalloprotease (gray enzyme) with an oxygen-(I)-anion and hydrogen-ion degrades the peptide bond. (3) When the substrate protein interacts with the enzyme, the zinc-(II)-cation binds to the carboxyl group of the substrate amino acid. The hydrogen of the enzyme bonds to the nitrogen of the amino acid, while the hydroxidion of the water molecule binds to the resulting free carbon. The remaining hydrogen binds to the oxygen-(I)-anion of the metalloprotease. (4) Degradation products. ChemDraw 21.0.0.28 (https://perkinelmerinformatics.com) was used for drawing, displaying, and characterizing chemical structures, substructures, and reactions. Biofilm and biological components were created with BioRender (https://www.biorender.com/)

Our secretome evaluation showed potential proteases in the supernatant of the *M. foliosa* enrichment culture. The fractionation of the supernatant and further LC-MS/MS analysis of two fractions resulted in the identification of a putative metalloprotease. Bacterial extracellular metalloproteases (BEMPs) cover families and subfamilies of serine or metalloproteases which degrade proteins outside of the bacterial cell (Wu and Chen 2011; Zhou et al. 2009). Furthermore, these endoproteases also play critical roles in bacterial virulence against eukaryotes (Wu and Chen 2011). Candidates of the family M4 are known to be involved in diseases caused by *P. aeruginosa*, *E. faecalis*, and *Vibrio cholerae* (Adekoya and Sylte 2009). These proteases destruct components of tissues and other cell compartments, like elastin, gelatin, and occludin (Yang et al. 2015; Wu et al. 2015; Francisco et al. 2021). The serratiopeptidase (serralysin) from *Serratia marcescens* belongs to the MEROPS family M10 and is responsible for lung and corneal damage (Nageswara et al. 2019). In addition, the zinc-dependent metalloprotease 1 (Zmp 1) produced by *Mycobacterium tuberculosis* was investigated for its role in human tuberculosis (TB) (Master et al. 2008; Liang et al. 2021). In general, the biofilm matrix is composed of extracellular polymeric substances like DNA, polysaccharides, proteins, and enzymes as well as lipids (Fig. 6A; Limoli et al. 2015; Majumdar et al. 2022; García et al. 2023) Metalloproteases act mainly on the degradation of proteins, depicted in Fig. 6B. For this, (endo)-metalloproteases hydrolyze the peptide bond between the amino acids. Mandatory for this is a water molecule linked to one or two zinc-(II)-cations connected to histidine amino groups of the enzyme. When the substrate enters the active site of the metalloprotease, the metal ions combine with the double-bonded oxygen of the carboxyl group of the target substrate protein. In the following reaction, the peptide bond is hydrolyzed, resulting in two separate amino acid chains (Hofmann 1985; Elsässer and Goettig 2021).

The enzyme assigned in this study could be comparable to ColA from *B. cereus*, a secreted collagenase known for gelatinolytic activity against native tropocollagen (Abfalter et al. 2016). It belongs to the MEROPS family M9 which possesses a Zn^2+^ in the active center and one activator domain, one peptidase domain, one or two polycystic kidney disease-like domains (PKD), and one to three collagen binding domains (CBD). This class of enzymes can degrade the major components in the extracellular matrix or on the cell surface in vertebrates, which makes these proteins potentially useful in pharmaceutical applications (Matsushita and Okabe 2001; Eckhard et al. 2013; Abfalter et al. 2016).

Interestingly, there is a series of pathogenic microorganisms, like *Vibrio alginolyticus* and *Streptococcus gordonii*, producing collagenases (Abfalter et al. 2016; Watanabe 2004). Predominantly, this group of metalloproteases attacks eukaryotic matrix components. In detail, these enzymes cleave important structural proteins of the EPS such as collagen and elastin. Further points of attack are the glycoproteins fibronectin, vitronectin, and entactin (Apte and Parks 2015; Mittal et al. 2016; Cui et al. 2017). However, some studies revealed that proteases, e.g., from the MEROPS family 23, can disrupt the cell walls of other bacteria and thus are suitable as an antimicrobial agent (Nilsen et al. 2003; Ahmed et al. 2003). Our microbial metalloprotease showed convincing activity against the biofilm formation of *S. maltophilia* K279a in an early phase. The confocal images validate impressively a detachment of cells and destruction of the dense film. The analysis of the live and dead cells proved a clear reduction of the biofilm thickness.

In summary, with the great potential of samples from the marine environment, we identified a bacterial metalloprotease with convincing biofilm prevention activity against a human pathogen. Possibly, it could be applied to prevent the successful attachment of biofilms of various strains in the medical field and thereby bring us closer to a solution to this serious challenge.

### Supplementary Information

Below is the link to the electronic supplementary material.

Supplementary file1 (PDF 2979 KB)

Supplementary file2 (XLSX 5761 KB)

## Data Availability

During this study, generated raw sequence data were deposited to the European Nucleotide Archive under the BioProject number PRJEB56465 for the (Meta)genome and transcriptome datasets. Raw datasets for the proteome analyses are deposit via PanoramaWeb (JobID 157735 and JobID 157734). Assemblies of the metagenome are available via IMG/MER (https://img.jgi.doe.gov) using the IMG ID 3300038501.
