# Supplementary material for: Novel marine metalloprotease—new approaches for inhibition of biofilm formation of Stenotrophomonas maltophilia
Source: Appl Microbiol Biotechnol. 2023 Sep 27;107(23):7119–34. doi: 10.1007/s00253-023-12781-0 (PMC10638167; doi:10.1007/s00253-023-12781-0)
Supplement: Supplementary file 1 — Supplementary file1 (PDF 2979 KB) [file 253_2023_12781_MOESM1_ESM.pdf]

Journal: Applied Microbiology and Biotechnology

Supplemental Material

Manuscript title: Novel marine metalloprotease – new approaches for inhibition of biofilm formation of *Stenotrophomonas maltophilia*

Authors: Marie Kristin Peters[1], Yekaterina Astafyeva[1], Yuchen Han[1], Jascha F.H. Macdonald[1], Daniela Indenbirken[2], Jacqueline Nakel[2], Sanamjeet Virdi[2], Guido Westhoff[3], Wolfgang R. Streit[1] and Ines Krohn[1]*

Affiliation(s) and address(es) of the author(s):

[1] University of Hamburg, Institute of Plant Science and Microbiology, Department of Microbiology and Biotechnology, Ohnhorststr.18, 22609 Hamburg, Germany

[2] Leibniz Institute of Virology, Technology Platform Next Generation Sequencing, Martinistraße 52, 20251 Hamburg, Germany

[3] Tierpark Hagenbeck, Gemeinnützige Gesellschaft mbH, Lokstedter Grenzstraße 2, 22527 Hamburg, Germany

***For Correspondence: Dr. Ines Krohn**

E-Mail: ines.krohn@uni-hamburg.de (https://orcid.org/0000-0001-8866-348X)

University of Hamburg, Institute of Plant Science and Microbiology,

Department of Microbiology and Biotechnology

Ohnhorststr.18,

D-22609 Hamburg, Germany,

Tel. (+49) 40-42816-444

Fax. (+49) 40-42816-459

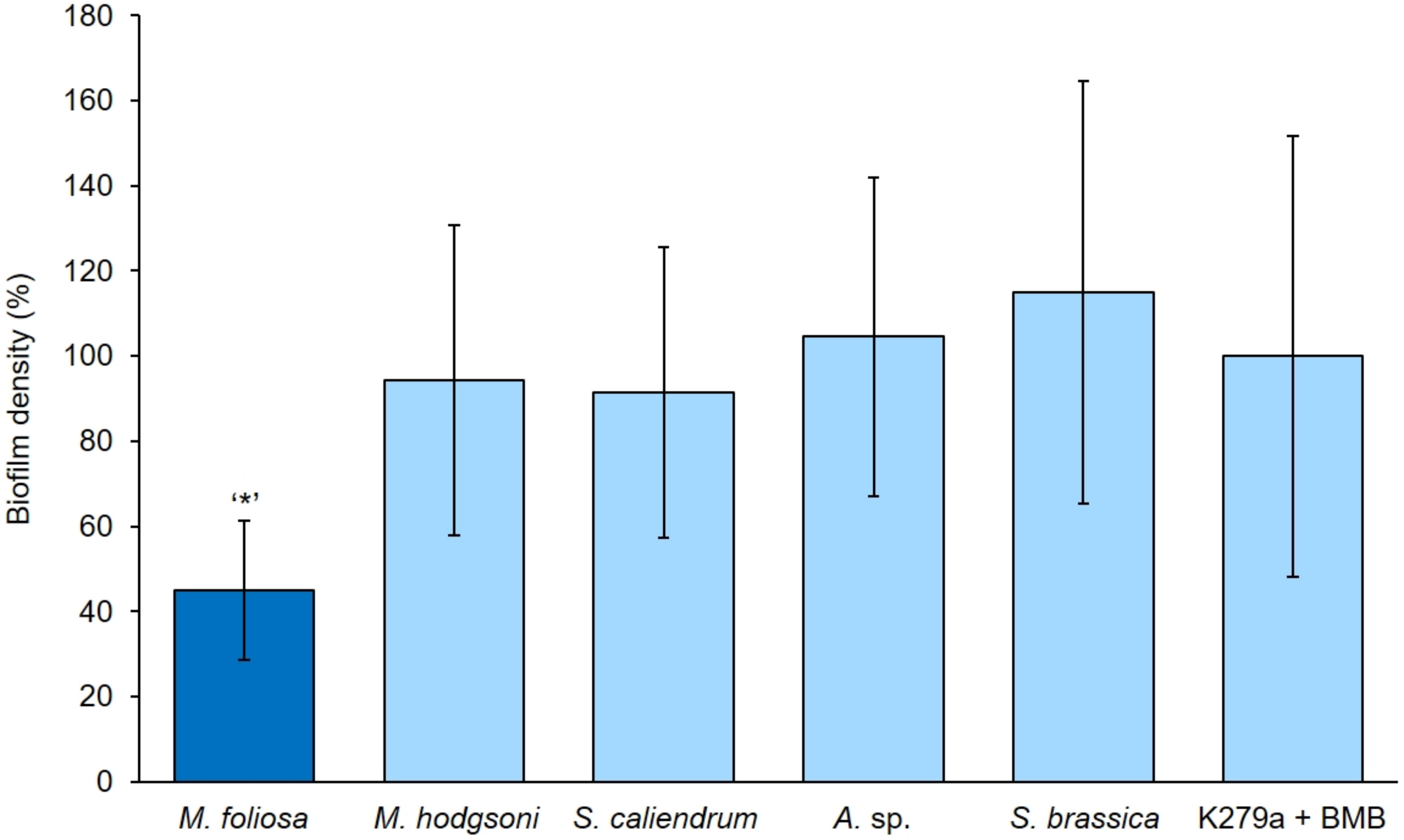

Supplemental Fig S1: Biofilm prevention assay with *S. maltophilia* K279a treated with supernatants of different enrichment cultures. The enrichment cultures were inoculated with parts of the following corals: *M. foliosa*, *M. hodgsoni*, *S. caliendrum*, *A.* sp., *S. brassica.* The K279a culture was incubated with the supernatants for 24 h in 96-well plates. The biofilm was stained with 0.5 % crystal violet and the density was measured at a wavelength of 595 nm. All samples were compared with the negative control *S. maltophilia* (K279a) treated with Bacto Marine Broth (BMB). Statistical analyses were subjected to a paired sample *t*-test and the *p*-value was referred to define if the two samples are significantly different from each other. Significant biofilm reduction marked by stars (significance level *p*-value ≤ 0.05). The sample with *M. foliosa* reduced the biofilm density significantly with a *p*-value of 1.3 x 10^-5. The data are mean values of at least three replicates. The error bars indicate simple standard deviations.

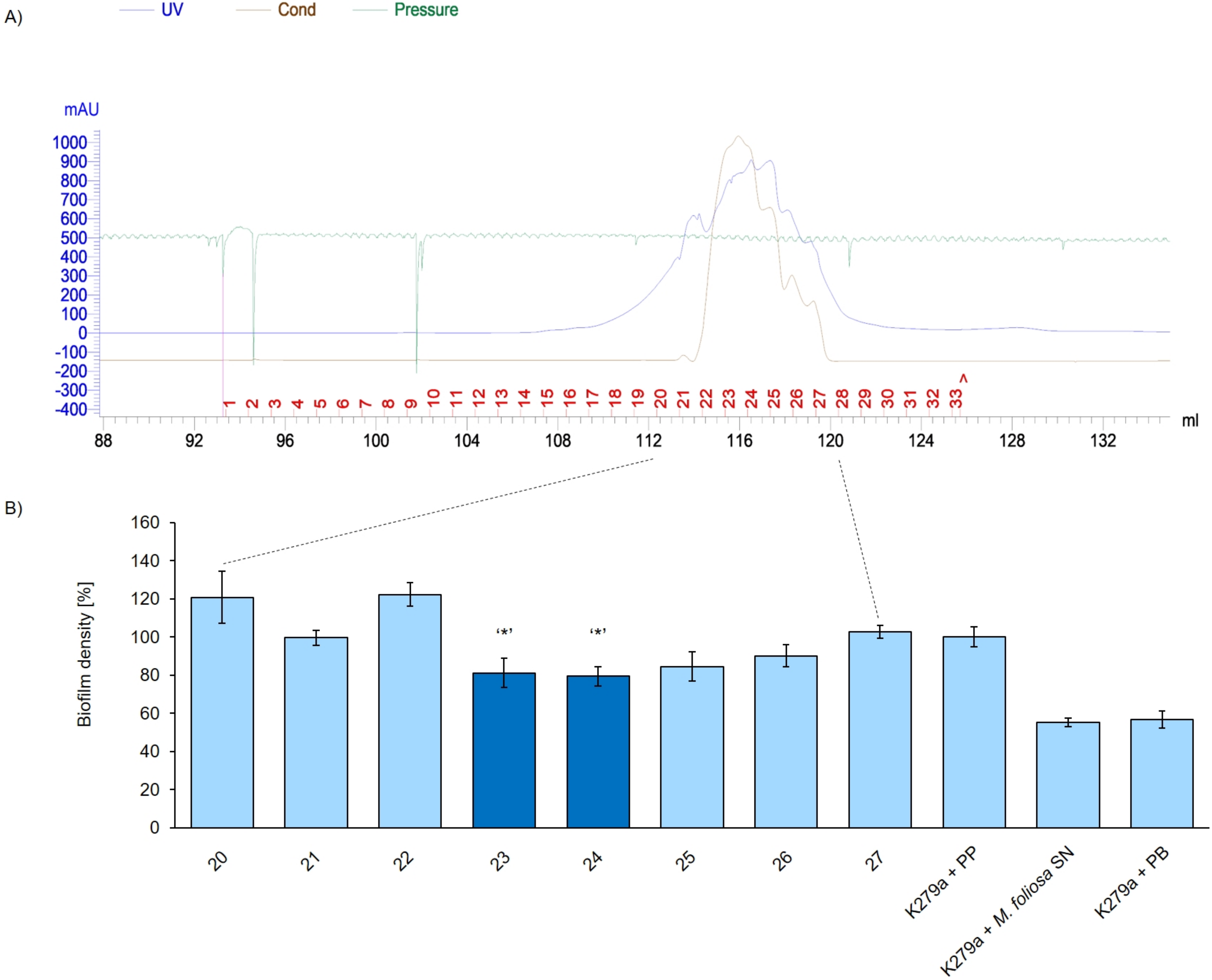

Supplemental Fig S2: A) Chromatogram of the *M. foliosa* culture after fractionation with the FPLC system and a Superdex® 200 10/300 GL gel filtration column. The supernatant was fractionated into 33 fractions. Potassium phosphate buffer pH 7.0 was the moving fluid. **B)** Biofilm prevention assay with *S. maltophilia* K279a treated with FPLC fractions of the *M. foliosa* enrichment culture. The K279a culture was incubated with the fractions for 24 h in 96-well plates. The biofilm was stained with 0.5 % crystal violet and the density was measured at a wavelength of 595 nm. 20 – 27: Fractions after FPLC clean-up. K279a+PP: *S. maltophilia* with potassium phosphate buffer. K279a+*M. foliosa* SN: *S. maltophilia* incubated with the whole supernatant of the *M. foliosa* culture. K279a+PB: Positive control, *S. maltophilia* treated with a protease from *Bacillus licheniformis*. Statistical analyses were subjected to a paired sample *t*-test and the *p*-value was referred to define if the two samples are significantly different from each other. Significant biofilm reduction marked by stars (significance level *p*-value ≤ 0.05). The fractions 23 and 24 significantly reduced the biofilm density with a *p*-value of 2.2 x 10^-4 and 5.6 x 10^-5, respectively. The data are mean values of at least three replicates. The error bars indicate simple standard deviations.

Supplemental Table S1: Used coral samples taken from the Hagenbeck Tropical Aquarium, and bacterial isolate.

Species	Characteristics/ origin
Montipora foliosa	Stony coral
Montipora hodgsoni	Stony coral
Seriatopora caliendrum	Stony coral
Acropora sp.	Stony coral
Sinularia brassica	Soft coral
Stenotrophomonas maltophilia K279a	Human blood, Bristol, UK, ATCC BAA-2423

Supplemental Table S2: Overall numbers of sequences and contigs generated for metagenome analysis of microbial communities from *M. foliosa*.

Parameter	Value
**Reads Illumina 1.9 (filtered)**	
Total sequence length (bp)	83,679,656
Duplicates (%)	37.45
GC (%)	42
**IMG ID metagenome statistic**	
IMG ID	3300038501
Number of bases	42,166,744
GC count [%]	48.39
Number of protein-coding genes	47,903
% of assembled protein-coding genes	98.87

Supplemental Table S3: Overall numbers of sequences and contigs generated for the transcriptome datasets of *S. maltophilia* K279a biofilm.

	**K279a control**	**K279a + *M. foliosa***
**Reads Illumina (filtered)**		
Total no.	8,072,838	8,895,226
Average length (bp)	147	149
Duplicates (%)	72.89	79.28
Fails (%)	25.76	28.79
GC (%)	57.33	62.50

Supplemental Table S5: Detected peptides in the supernatant of the enrichment culture with *M. foliosa* using electrospray ionization mass spectrometry coupled with liquid chromatography (filtered data).

Accession (NCBI)	Number of peptides	Avg. mass (DA)	Description
P68734|NPRE_BACPU	32	32674	Neutral protease NprE OS=*Bacillus pumilus*
P68736|NPRE_BACSU	32	56522	Bacillolysin OS=*Bacillus subtilis* (strain 168)
P68735|NPRE_BACSA	32	56522	Bacillolysin OS=*Bacillus subtilis* subsp. *amylosacchariticus*
P00691|AMY_BACSU	25	72378	Alpha-amylase OS=*Bacillus subtilis* (strain 168)
P54422|GGT_BACSU	24	64189	Glutathione hydrolase proenzyme OS=*Bacillus subtilis* (strain 168)
P04189|SUBT_BACSU	16	39479	Subtilisin E OS=*Bacillus subtilis* (strain 168)
P00783|SUBT_BACSA	15	39467	Subtilisin amylosacchariticus OS=*Bacillus subtilis* subsp. *amylosacchariticus*
P29142|SUBT_GEOSE	15	39495	Subtilisin J OS=*Geobacillus stearothermophilus*
P39790|MPR_BACSU	13	33842	Extracellular metalloprotease OS=*Bacillus subtilis* (strain 168)
P63186|GGT_BACNA	19	64070	Glutathione hydrolase proenzyme OS=*Bacillus subtilis* subsp. *natto*
O07921|CHIS_BACSU	14	31497	Chitosanase OS=*Bacillus subtilis* (strain 168)
P24141|OPPA_BACSU	12	61525	Oligopeptide-binding protein OppA OS=*Bacillus subtilis* (strain 168)
P39116|PLY_BACSU	12	45498	Pectate lyase OS=*Bacillus subtilis* (strain 168)
Q45070|XYNC_BACSU	12	47337	Glucuronoxylanase XynC OS=*Bacillus subtilis* (strain 168)
P25152|BSAP_BACSU	17	49450	Aminopeptidase YwaD OS=*Bacillus subtilis* (strain 168)
P54423|WPRA_BACSU	17	96488	Cell wall-associated protease OS=*Bacillus subtilis* (strain 168)
P29141|SUBV_BACSU	13	85608	Minor extracellular protease vpr OS=*Bacillus subtilis* (strain 168)
O34313|NTPES_BACSU	18	159705	Trifunctional nucleotide phosphoesterase protein YfkN OS=*Bacillus subtilis* (strain 168)

Supplemental Table S6: Most abundant proteins in the active fractions 23 and 24 of the enrichment culture supernatant with *M. foliosa*. Based on a proteome analysis using electrospray ionization mass spectrometry coupled with liquid chromatography (filtered data).

**Accession (UniProt)**	**Number of sequences**	**Mass (DA)**	**Sequence coverage**	**Description**
A0A1J9WHY4	68	100256	0.74	Microbial collagenase OS=*Bacillus cereus*
A0A0G3B7J6	10	110008	0.13	Microbial collagenase OS=*Bacillus cereus*
A0A2B2M9I7	4	109685	0.07	Microbial collagenase OS=*Bacillus cereus*
A0A1J9WEP7	13	63716	0.28	Peptide ABC transporter substrate-binding protein OS=*Bacillus cereus*
A0A1Y6A081	3	63395	0.06	Peptide ABC transporter substrate-binding protein OS=*Bacillus cereus*
A0A0J7GMX8	3	120049	0.04	Collagen-binding protein OS=*Bacillus cereus*
